# Usefulness of C-reactive protein in monitoring the severe community-acquired pneumonia clinical course

**DOI:** 10.1186/cc6105

**Published:** 2007-08-28

**Authors:** Luís Coelho, Pedro Póvoa, Eduardo Almeida, Antero Fernandes, Rui Mealha, Pedro Moreira, Henrique Sabino

**Affiliations:** 1 Unidade de Cuidados Intensivos, Hospital Garcia de Orta, Almada, Portugal

## Abstract

**Background:**

The aim of the present study was to evaluate the C-reactive protein level, the body temperature and the white cell count in patients after prescription of antibiotics in order to describe the clinical resolution of severe community-acquired pneumonia.

**Methods:**

A cohort of 53 consecutive patients with severe community-acquired pneumonia was studied. The C-reactive protein levels, body temperature and white cell count were monitored daily.

**Results:**

By day 3 a C-reactive protein level 0.5 times the initial level was a marker of poor outcome (sensitivity, 0.91; specificity, 0.59). Patients were divided according to their C-reactive protein patterns of response to antibiotics, into fast response, slow response, nonresponse, and biphasic response. About 96% of patients with a C-reactive protein pattern of fast response and 74% of patients with a slow response pattern survived, whereas those patients with the patterns of nonresponse and of biphasic response had a mortality rate of 100% and 33%, respectively (*P *< 0.001). On day 3 of antibiotic therapy, a decrease in C-reactive protein levels by 0.31 or more from the previous day's level was a marker of good prognosis (sensitivity, 0.75; specificity, 0.85).

**Conclusion:**

Daily C-reactive protein measurement after antibiotic prescription is useful in identification, as early as day 3, of severe community-acquired pneumonia patients with poor outcome. The identification of the C-reactive protein pattern of response to antibiotic therapy was useful in the recognition of the individual clinical course, either improving or worsening, as well as the rate of improvement, in patients with severe community-acquired pneumonia.

## Introduction

Community-acquired pneumonia (CAP) remains a common and serious illness, with an estimated incidence of 2–12 cases/1,000 population per year [1]. The majority of cases are managed outside hospital, but approximately 20% require hospital admission. Out of this group of patients, around 10% develop severe CAP [2] requiring treatment in an intensive care unit (ICU) with a mortality rate exceeding 50% [1,3]. The largest numbers of deaths occur in the first few days of hospitalization [4], so the early recognition of patients with severe CAP not only aids in the early initiation of antibiotic therapy but also in adequate supportive care.

It has been estimated that approximately 10–25% of patients with CAP do not resolve within the anticipated time [5]. Treatment failure can result from a lack of response by the host or from the development of an infectious complication, such as postobstructive pneumonia, empyema, or lung abscess. In addition, treatment failure may be wrongly presumed when radiologic infiltrates are resolving slowly but the patient has a superimposed problem, such as drug fever, malignancy, inflammatory conditions, heart failure, or a hospital-acquired infection from another source [3]. In such clinical situations, it is very difficult to identify the cause of the presumed treatment failure, since clinical and radiological evaluation is insufficient to differentiate an infectious complication from a noninfectious complication. Some studies [6,7] evaluated the value of some serum markers of infection, such as C-reactive protein (CRP) and interleukins, in monitoring the response to antibiotic treatment. In the present study we hypothesize that daily monitoring of plasma CRP can recognize patients with bad outcome and patients with good outcome early in the course of antibiotic treatment.

Plasma CRP is an acute phase-protein synthesized only by the liver largely under transcriptional control of IL-6 [8]. CRP levels rise rapidly in response to several inflammatory stimuli, bacterial infection being one of the most potent. The secretion of CRP begins within 4–6 hours of the stimulus, doubling every 8 hours, and peaking at 36–50 hours. After the disappearance of or removal of the stimulus, the CRP concentration decreases rapidly with a half-life of 19 hours [9].

The aim of the present study was to assess the value of serial CRP determinations after prescription of antibiotics in the evaluation of the resolution of severe CAP, in order to recognize, early in the clinical course, patients with good outcome and patients with bad outcome, as well as to identify the individual patterns of the CRP response to antibiotics.

## Materials and methods

### Study subjects

A prospective observational cohort study was conducted between November 2001 and December 2002 in the ICU of Garcia de Orta Hospital (Almada, Portugal). All patients who were aged ≥18 years and admitted for severe CAP were enrolled. The Ethics Committee of Garcia de Orta Hospital approved the study design; informed consent was waived as there was no need for additional blood samples.

### Study design

The data collected included the admission diagnosis, the past medical history and vital signs. The CRP concentration, the body temperature, the white cell count (WCC), the Sequential Organ Failure Assessment (SOFA) score [10,11] and the PaO_2_/FiO_2 _ratio were recorded daily. After clinical CAP diagnosis, all patients received empirical antibiotic therapy according to the American Thoracic Society CAP guidelines [2].

For the purposes of time-dependent analysis, day 0 was defined as the day of CAP clinical diagnosis. The following days were successively defined as day 1, day 2, and so on.

Withdrawal of the inflammatory stimulus results in a sharp decrease in the serum CRP concentration, similar to first-order elimination kinetics [8]. As a result, time-dependent analysis of the relative CRP concentration (CRP ratio) was also performed. The CRP ratio was calculated in relation to the day 0 CRP concentration. The maximal relative CRP variation from the previous day's CRP level was also analysed.

Patients were followed-up until pneumonia was cured or until death. The progression of the CRP concentration, the CRP ratio, the body temperature and the WCC throughout the course of severe CAP was analysed, comparing survivors with nonsurvivors.

### Definitions

Severe CAP was defined according American Thoracic Society guidelines [3]. Previous antibiotic treatment was defined as any antibiotic treatment in the week before ICU admission. Adequate antibiotic therapy was defined, in the empirical therapy prescribed by the onset of severe CAP, as at least one antibiotic covering all of the pathogens isolated, as determined by the sensitivity pattern in the antibiogram. In patients started with initially inadequate treatment, antibiotics were changed according to the pathogen isolated and according to antimicrobial susceptibility testing.

Patients were retrospectively classified according to previously defined CRP patterns of the response to antibiotic [12,13]: fast response occurred when the CRP ratio at day 4 was <0.4 relative to the day 0 CRP; slow response was characterized by a continuous and slow decrease in the CRP ratio; nonresponse was when the CRP ratio remained ≥0.8; and biphasic response was characterized by an initial CRP ratio decrease to levels <0.8 followed by a secondary rise to values ≥0.8. CAP patients were retrospectively divided into four groups according to their pattern of CRP response.

### Analysis

Continuous variables are presented as the mean ± standard deviation, unless stated otherwise. The Shapiro–Wilk test was used for normality assessment. Comparisons between groups were performed using the parametric unpaired and paired *t*-test, or the nonparametric Mann–Whitney U-test and the Wilcoxon signed-rank test for continuous variables according to data distribution. The chi-squared test was used to carry out comparisons between categorical variables. Time-dependent analysis of different variables was performed via general linear model univariate repeated-measures analysis using a split-plot design approach.

Receiver-operating characteristic curves were drawn for the CRP ratio, the body temperature and the WCC on day 3 of antimicrobial therapy. The indicative accuracy of these variables at day 3 was assessed by calculation of the area under the curve (AUC), as described elsewhere [14]. In medical practice, a diagnostic test with an AUC <0.75 is regarded as noncontributive [15]. Comparison of the AUC of two variables was performed using the method of Hanley and McNeil [16]. Results are reported with the 95% confidence interval. Significance was accepted at *P *< 0.05.

## Results

During the study period, 53 patients were admitted to the ICU with severe CAP. Of these 53 patients, 13 (24.5%) died in the ICU, all deaths occurring while patients were still on antibiotic treatment and were mechanically ventilated. Fourteen patients (26.4%) were already receiving empiric antibiotic treatment on ICU admission; all patients maintained the antibiotic treatment already prescribed. The microbiological diagnosis was established in 11 patients (21%). All patients with microbiological diagnosis had initial adequate antibiotic treatment; only one patient with initial adequate antibiotic therapy died. Five patients (9.4%) were on corticosteroid treatment on ICU admission for chronic obstructive pulmonary disease exacerbation. The demographic characteristics of the patients with severe CAP are presented in Table 1. On ICU admission, 91% of patients were already mechanically ventilated.

**Table 1:** Characteristics of the patient population with severe community-acquired pneumonia

	Survivors (*n *= 40)	Nonsurvivors (*n *= 13)	*P *value
Age (years)	59.4 ± 14.8	61.1 ± 12.1	0.220
Sex (male/female)	31/9	8/5	0.257
C-reactive protein day 0 (mg/dl)	23.6 ± 18.4	23.9 ± 11.6	0.591
Acute Physiology, Age, and Chronic Health Evaluation II score	17.8 ± 5.7	26.1 ± 6	<0.001
Sequential Organ Failure Assessment score day 0	6.5 ± 2.5	9.7 ± 2.9	0.002

Data presented as the mean ± standard deviation.

**Figure 1:**
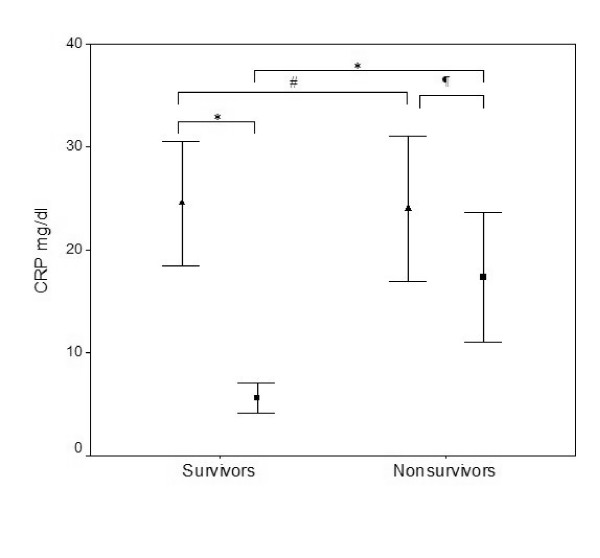
C-reactive protein levels. C-reactive protein (CRP) levels on the day of antibiotic prescription (▲, day 0) and on the last day of antibiotic therapy in survivors or at death in nonsurvivors (■). Data presented as the mean ± standard deviation. ^#^*P *= 0.591. ^¶^*P *= 0.021. *P < 0.001.

**Figure 2:**
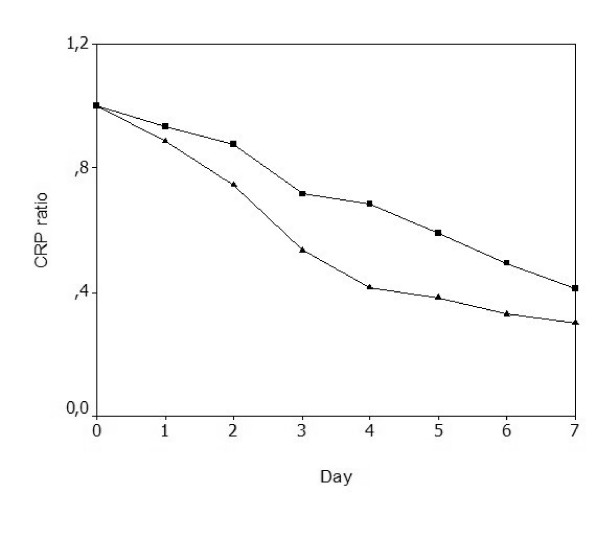
Time-dependent analysis of the C-reactive protein ratio during antibiotic therapy. Time-dependent analysis of the C-reactive protein (CRP) ratio during antibiotic therapy, from day 0 to day 7 of antibiotic therapy, was significantly different between survivors (▲) and nonsurvivors (■). *P *= 0.039.

At day 0, the CRP concentration, the body temperature and the WCC of survivors and nonsurvivors were not significantly different: 23.6 ± 18.4 mg/dl versus 23.9 ± 11.6 mg/dl (*P *= 0.591) (Figure 1), 38.0 ± 0.75°C versus 37.9 ± 1.1°C (*P *= 0.856) and 16.2 ± 13.9 × 10^3 ^cells/μl versus 13.9 ± 12.7 × 10^3 ^cells/μl (*P *= 0.227), respectively. From day 0 to day 7 of antibiotic therapy, time-dependent analysis of the CRP ratio in survivors showed a more steady and significant decrease than that in nonsurvivors (*P *= 0.039) (Figure 2). Over the same time period, the body temperature decreased likewise in both groups (*P *= 0.249). Analysis of the WCC showed no differences between survivors and nonsurvivors (*P *= 0.423).

At day 3, the CRP ratio in survivors was 0.49 relative to the initial level (*P *< 0.001), whereas in nonsurvivors the CRP ratio remained elevated at 0.71 (*P *= 0.002). The AUC for the CRP ratio by day 3 was 0.76 (95% confidence interval = 0.61–0.87), whereas the AUCs of the WCC and the body temperature by day 3 were 0.45 (95% confidence interval = 0.25–0.65) and 0.44 (95% confidence interval = 0.24–0.64), respectively. The AUC of the CRP ratio by day 3 was significantly greater than that of the WCC and the body temperature (*P *= 0.022 and *P *= 0.047, respectively). A CRP ratio >0.5 of the day 0 concentration by day 3 was a marker of poor outcome, with a sensitivity of 0.91, a specificity of 0.55, a negative predictive value of 0.95 and a positive predictive value of 0.4 (positive likelihood ratio, 6.05; negative likelihood ratio, 0.49).

At the end of antibiotic therapy, the CRP concentration of survivors was 5.4 ± 4.2 mg/dl. In nonsurvivors, on the day of death the CRP concentration increased from the day 7 value, reaching 16.3 ± 8.8 mg/dl (*P *< 0.001). The body temperature at the end of antibiotic therapy in survivors was similar to that in nonsurvivors on the day of death (37.1 ± 0.9°C and 37.5 ± 0.7°C, respectively; *P *= 0.60) and the WCC was not significantly different (11.1 ± 5.0 × 10^3 ^cells/μl versus 16.3 ± 9.8 × 10^3 ^cells/μl, respectively; *P *= 0.165). Only survivors showed a significant decrease in body temperature (*P *< 0.001).

Patients with severe CAP were retrospectively divided according to four patterns of the CRP ratio course during antibiotic therapy. Twenty-two patients were classified as fast response, 23 patients as slow response, five patients as nonresponse and three patients as biphasic response. Time-dependent analysis of the CRP ratio of the four different patterns showed that these patterns of progression were significantly different (*P *< 0.001). By day 3, the CRP ratio was 0.31 ± 0.10, 1.30 ± 1.50, 0.90 ± 0.26 and 0.97 ± 0.27 in patients exhibiting a fast response, a slow response, nonresponse and a biphasic response pattern, respectively (*P *< 0.001). Conversely, during the same time period, no significant difference between the different patterns was found in the progression of the WCC and the body temperature (*P *= 0.731 and *P *= 0.152, respectively).

We then went on in our analysis to study the correlation between the CRP ratio patterns and the outcome. About 96% of patients with a CRP ratio pattern of fast response and 74% of patients with a slow response pattern survived, whereas those patients with the patterns of nonresponse and biphasic response exhibited overall mortality rates of 100% and 33%, respectively (*P *< 0.001). Together, the combined mortality rate of patients with these two latter patterns was 75%.

**Figure 3:**
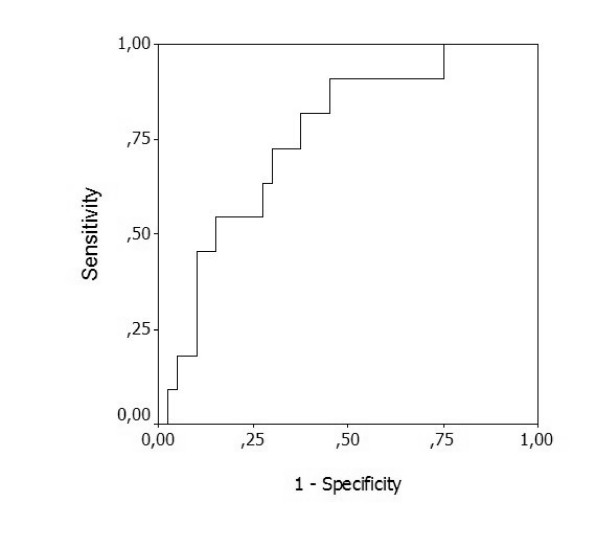
Maximal daily C-reactive protein variation. Receiver-operating characteristics curve of the maximal daily C-reactive protein variation from the level of the previous day. Area under the curve, 0.76 (95% confidence interval = 0.61–0.86).

We analysed the maximal daily relative CRP concentration variation from the previous day's level between day 0 and the last day of antibiotic therapy. The receiver-operating characteristic curve AUC for maximal daily relative CRP variation was 0.76 (95% confidence interval = 0.61–0.86) (Figure 3). A decrease in CRP levels by 0.31 or more from the previous day's concentration was a marker of good prognosis (sensitivity, 0.75; specificity, 0.85; positive likelihood ratio, 4.87; negative likelihood ratio, 0.30; negative predictive value, 0.92; positive predictive value, 0.61).

During antibiotic therapy, 29 out of 53 patients with severe CAP had, at least once, a relative CRP variation from the previous day's level ≥0.31. Out of these 29 patients, 27 were survivors and two patients were nonsurvivors (*P *= 0.001); in addition, in one-half of the patients this variation took place in the first 3 days of antibiotic therapy. By day 3, 90% of severe CAP patients with a fast response pattern had had at least one relative CRP variation from the previous day's level of 0.31 or more, whereas this was observed in only 60% of patients with a pattern of slow response.

Clinical progression during antibiotic therapy was monitored with daily measurement of the SOFA score and the PaO_2_/FiO_2 _ratio. The result of time-dependent analysis of the PaO_2_/FiO_2 _ratio from day 0 to day 7 of antibiotic therapy in survivors and nonsurvivors was not significantly different (*P *= 0.339). Moreover, the same analysis of the PaO_2_/FiO_2 _ratio for the four different CRP ratio patterns from day 0 to day 7 showed no significant differences between the patterns (*P *= 0.229).

During the same period, the SOFA score progression between survivors and nonsurvivors was significantly different (*P *= 0.013). The assessment of the SOFA score progression according to the four different CRP ratio patterns, however, showed no differences (*P *= 0.142).

## Discussion

In the present study, we monitored the clinical resolution of severe CAP after institution of antibiotic therapy assessed by serial measurements of the CRP concentration, the body temperature and the WCC, in order to identify, early in the clinical course, patients with good outcome and patients with bad outcome.

The evaluation of clinical resolution of CAP is presently based on the daily assessment of the same parameters used in diagnosis, namely X-ray scan, body temperature and WCC. Most of these parameters are unspecific, however, and can be influenced by factors not related to CAP itself. In addition, the radiological resolution often lags behind the clinical improvement from CAP, so it is not a useful tool to predict outcome [2,17,18].

The use of biomarkers to estimate the presence of an infection and its treatment response is not well studied in CAP patients. Several studies have shown that CRP is a good marker of CAP diagnosis, as well as useful for assessing its clinical severity [19-21]. Other markers, such as procalcitonin, have proved to be good predictors of complications and mortality [22].

Smith and colleagues studied 28 CAP patients after the prescription of antibiotics, from day 1 until day 5 of therapy, assessing the serial changes of the plasma CRP, tumour necrosis factor alpha and IL-6 [7]. In that study, on the day of CAP diagnosis all patients presented high CRP levels, >5 mg/dl. Another interesting finding was that the admission CRP concentration was significantly influenced by the antibiotic prescription prior to hospital admission in comparison with those patients without therapy (10.7 ± 4.2 versus 15.2 ± 4.4, respectively; *P *= 0.023). The authors showed that in patients with a good outcome the CRP concentration fell sharply, whereas in patients who died of pneumonia there was a progressive rise in the CRP level prior to death, to concentrations >10 mg/dl. We found in our study a similar CRP course in survivors and nonsurvivors. The other biomarkers studied by Smith and colleagues were not helpful in the assessment of the CAP clinical course. Tumour necrosis factor alpha was detectable in only six patients on the day of hospital admission, and only a further seven patients had detectable concentrations during the period of follow-up. Concerning IL-6, only six patients had detectable concentrations during some point of their hospital stay.

In a previous study, our group assessed the value of daily measurements of CRP, WCC and body temperature after the prescription of antibiotics in ventilator-associated pneumonia patients [12]. In that study, daily CRP measurements after antibiotic prescription were useful in the identification, as early as day 4, of ventilator-associated pneumonia patients with poor outcome. Moreover, both the WCC and the body temperature were not useful early markers of the ventilator-associated pneumonia course. Patients were also divided according to the pattern of CRP response to antibiotics; all patients with fast and slow response patterns survived, whereas those patients showing nonresponse and a biphasic response pattern exhibited a mortality of 78% and 75%, respectively. The influence of adequate initial antibiotic therapy on the outcome of ventilator-associated pneumonia patients was also studied. Patients with inadequate initial antibiotic therapy had a mortality rate of 66.7%, while patients with adequate therapy showed mortality of 18.4%.

In the present study, serial measurements of the CRP concentration, the body temperature and the WCC were performed in patients with severe CAP from the day of antibiotic prescription (day 0) to the day of death or to the end of antibiotic therapy, dividing patients into survivors and nonsurvivors. Daily CRP measurements were performed not to predict outcome but to describe the clinical course. From day 0 to day 7 the CRP ratio showed a significant and steady decrease in survivors, whereas in nonsurvivors it remained elevated. In survivors, by day 3 the CRP ratio had decreased by almost 50% from the admission concentration. Comparisons of receiver-operating characteristic curves showed that the prognostic performance of the CRP ratio by day 3 was significantly better than that of the body temperature and the WCC. A CRP ratio >0.5 by day 3, with a sensitivity of 0.91 and a specificity of 0.55, was associated with the diagnosis of nonresolving severe CAP.

We additionally performed the analysis of the maximal relative variation of CRP from the previous day's level. We found that a decrease higher than 0.31 from the previous day was a marker of good prognosis, with an AUC of 0.76, a sensitivity of 0.75 and a specificity of 0.85. Almost 80% of survivors showed a decrease higher than 0.31. In addition, the rate of the CRP decrease expressed by the maximal relative CRP variation from the previous day's level had a good correlation with a good clinical course.

The CRP ratio patterns of patient response to antibiotics were found to be closely correlated with outcome. About 76% of patients with fast and slow response patterns survived, whereas the combined mortality rate of the patients showing the nonresponse and biphasic response patterns was 75%.

The optimal duration of antibiotic therapy in CAP is still unknown, and possibly should vary from patient to patient depending of the severity of the pneumonia as well as the clinical course. Current guidelines recommend antibiotic courses from 7 to 21 days, depending on the pneumonia severity and the type of pathogen [2,3]. In a recent published study, Christ-Crain and colleagues proposed procalcitonin to diagnose and guide the duration of antibiotic therapy in CAP patients. Patients in the procalcitonin guidance group reduced their antibiotic therapy duration to 5 days, compared with 12 days in patients treated according with guidelines [23]. Twenty-nine per cent of the patients included in this study, however, had an almost undetectable level of procalcitonin on the day of diagnosis. Consequently, in those patients it is virtually impossible to evaluate the rate of procalcitonin decline since it is already very low. As a result, procalcitonin can hardly be a valuable marker to guide the duration of antibiotic therapy or to predict outcome at least in patients that were diagnosed as CAP but had unexpectedly very low procalcitonin levels.

The evaluation of changes in clinical variables, such as the SOFA score and the PaO_2_/FiO_2 _ratio, can be helpful in the assessment of the effect of different therapeutic interventions [24]. In this study, the PaO_2_/FiO_2 _ratio did not discriminate between survivors and nonsurvivors during the first week of antibiotic therapy, confirming the data published previously for ventilator-associated pneumonia patients [12]. This ratio parameter depends profoundly on noninfectious factors and can be easily influenced, for example, by the FiO_2 _administered or by the ventilator settings.

Conversely, a significant decrease in the SOFA score from day 0 to day 7 was found in survivors, whereas in nonsurvivors the values remain almost unchanged. Patients with good outcome had a progressive decrease in the CRP ratio, showing a good correlation with the resolution of organ failure measured by the SOFA score. Lobo and colleagues [24] found that increased CRP concentrations were associated with organ failure, prolonged ICU stay and high infection and mortality rates. Increasing or persistently high levels (suggesting ongoing inflammatory activity) indicated poor prognosis, while declining values (suggesting a diminishing inflammatory reaction) were associated with a more favourable prognosis. In our study, patients who maintained high levels of CRP, suggesting a persistent inflammatory response – namely those with nonresponse and biphasic response patterns of response – had significantly higher SOFA scores as well as higher mortality rates. On the contrary, patients who presented progressively declining levels of CRP showed a SOFA score improvement and a better prognosis. The SOFA score is not a sepsis-related score as the authors initially thought, however, but just an organ failure/dysfunction score [10,11]. Consequently, the SOFA score can be influenced by several noninfectious conditions unrelated to the course of the primary infection.

We should note some limitations of the present investigation. The study is a cohort, single-centre, observational study using variables collected daily at the bedside to evaluate the clinical course of severe CAP. We should note that this issue was only fully addressed in a very limited number of studies, however – and the CRP concentration used in only one other study [7] – so it is very difficult to compare results.

## Conclusion

In summary, it has been demonstrated that daily CRP measurements after prescription of antibiotic therapy are useful in the identification, as early as day 3, of severe CAP patients with poor outcome, and the measurement performs better than the commonly used markers of infection, such as body temperature and WCC. In addition, recognition of the pattern of the CRP ratio response to therapy could provide more information about the individual clinical course improving or worsening, as well as the rate of improvement. In addition, our data suggest that, in patients with severe CAP with a rapid CRP ratio decline, a shorter duration of antibiotic therapy could be equally effective, reducing toxicity, reducing the risks of emergence of resistant strains and reducing costs. Conversely, for patients showing the patterns of nonresponse and biphasic response, we should perform an aggressive diagnostic and therapeutic approach to prevent further clinical worsening. If these findings are confirmed, the duration of antibiotic therapy could be tailored to each patient's clinical response, and CRP can be an important marker in daily monitoring for the efficacy of antibiotic therapy of patients with severe CAP. Further studies to assess the clinical impact of daily monitoring should be performed.

## Key messages

• Daily CRP measurement is useful in monitoring the clinical course of severe CAP and is a good early marker of favourable outcome.

• The rate of CRP decrease expressed by the maximal relative CRP variation from the previous day's level has a good correlation with a good clinical course.

• The identification of the pattern of the CRP response to antibiotic therapy might be useful in the recognition of the individual clinical course either improving or worsening in patients with severe CAP, as well as the rate of improvement.

• Daily CRP ratio measurements and the patterns of the CRP response to antibiotics have a good correlation with the clinical course assessed by the SOFA score in patients with severe CAP.

## Abbreviations

AUC = area under the curve; CAP = community-acquired pneumonia; CRP = C-reactive protein; FiO_2 _= fractional inspired oxygen; ICU = intensive care unit; IL = interleukin; PaO_2 _= arterial oxygen tension; SOFA = Sequential Organ Failure Assessment; WCC = white cell count.

## Competing interests

The authors declare that they have no competing interests.

## Authors' contributions

LC and PP conceived the study. All authors participated in the original design and in writing the original protocol. LC and PP collected and analysed the data and drafted the manuscript. All authors read and approved the final manuscript.
